# New trends in brain tumor immunity with the opportunities of lymph nodes targeted drug delivery

**DOI:** 10.1186/s12951-023-02011-0

**Published:** 2023-08-04

**Authors:** Yangzhi Qi, Wei Xiong, Qianxue Chen, Zhifei Ye, Cailei Jiang, Yan He, Qingsong Ye

**Affiliations:** 1 Center of Regenerative Medicine, Department of Stomatology, Renmin Hospital of Wuhan University, Gaoxin 6th Road, Jiangxia, Wuhan, 430000 Hubei People’s Republic of China; 2 Department of Neurosurgery, Renmin Hospital of Wuhan University, Wuhan, 430060 Hubei China; 3 Clinical Research Center, The Second Linhai Renmin Hospital, Linhai, 317000 Zhejiang China; 4 Institute of Translational and Regenerative Medicine, Tianyou Hospital, Wuhan University of Science and Technology, Wuhan, 430040 Hubei China; 5 Department of Oral and Maxillofacial Surgery, Massachusetts General Hospital, Harvard Medical School, Boston, MA 02114 USA

**Keywords:** Lymph nodes, Targeted drug delivery, Glioblastoma, Nanoparticles, Meningeal lymphatic vessels, Tumor immunity

## Abstract

Lymph nodes targeted drug delivery is an attractive approach to improve cancer immunotherapy outcomes. Currently, the depth of understanding of afferent and efferent arms in brain immunity reveals the potential clinical applications of lymph node targeted drug delivery in brain tumors, e.g., glioblastoma. In this work, we systematically reviewed the microenvironment of glioblastoma and its structure as a basis for potential immunotherapy, including the glial-lymphatic pathway for substance exchange, the lymphatic drainage pathway from meningeal lymphatic vessels to deep cervical lymph nodes that communicate intra- and extracranial immunity, and the interaction between the blood–brain barrier and effector T cells. Furthermore, the carriers designed for lymph nodes targeted drug delivery were comprehensively summarized. The challenges and opportunities in developing a lymph nodes targeted delivery strategy for glioblastoma using nanotechnology are included at the end.

## Background

The lymph nodes (LNs) are an important transit structure for lymphatic vessels and an important part of the lymphatic system [1]. LNs gather immunogenic information from periphery for presentation to antigen present cells (APC), where lymphocytes are activated to provide protective immunity in the periphery [2]. Thus, delivering drugs directly to lymph nodes may provide an opportunity to induce local and systemic responses in diseases, e.g., in various types of advanced cancers with a predilection for metastasis [3]. However, LNs targeted drug delivery has not achieved success in treating brain tumors so far. Recently, a series of studies have reported functional lymphatic vessels in central nervous system (CNS), highlighting the potential clinical significance of LNs targeted drug delivery in brain tumor therapy [4, 5].

The management of glioblastoma (GBM), the deadliest malignant brain tumor, has proven to be extremely challenging in the past decades [6]. Despite treatment combining surgery and adjuvant radio-chemotherapy, patients’ outcomes remain poor, with a 5-year overall survival rate of 5.4% [6]. Activation of immune functions in the brain and/or allowing chemotherapeutic agents to cross the blood–brain barrier (BBB) become the focus of GBM treatment. The discovery of lymphatic vessels and the glial-lymphatic pathway in the brain and their communication with cervical LNs has greatly completed the immune afferent and efferent arms in CNS [4, 5, 7, 8]. These findings imply that LNs targeted delivery may not only activate immune signaling directly but also provide a route for drugs to bypass the BBB and enter the CNS. Notably, a new tumor vaccine using dendritic cells (DCs) as carriers to target LNs to activate peripheral immunity, DCVax-L vaccine, has significantly improved patients’ overall survival in both newly diagnosed and recurrent GBM according to a recent phase 3 clinical trial, which has never been achieved in any other comparative immunotherapeutic strategies [9]. Moreover, a recent study also revealed that drugs injected from cervical lymph nodes can reach brain along lymph vessels [10]. That means LNs targeted drug delivery acts as the main means of tumor vaccine delivery as well as a smart way of drug delivery to bypass the BBB, which possesses great potential in the future treatment of GBM. On the other hand, there is a lack of experimental and clinical evidence for LNs targeted delivery for brain tumors. Therefore, it is of significance to review the structure rationale, and current advances in LNs targeted drug delivery of brain tumors to facilitate the clinical translation of related therapeutics.

Herein, we reviewed the immunity of the brain, and deeply discussed its structure and the functions of its components. The afferent and efferent arms of brain immune system were systematically reviewed. Then the carriers designed for LNs targeted drug delivery are carefully surveyed. At the end, the challenges and opportunities of LNs targeted delivery strategy in GBM are proposed.

## The afferent and efferent arms of immune system and their structure basis in brain

### The discovery of meningeal lymphatic network and cerebral spinal fluid circulation

**Fig. 1:**
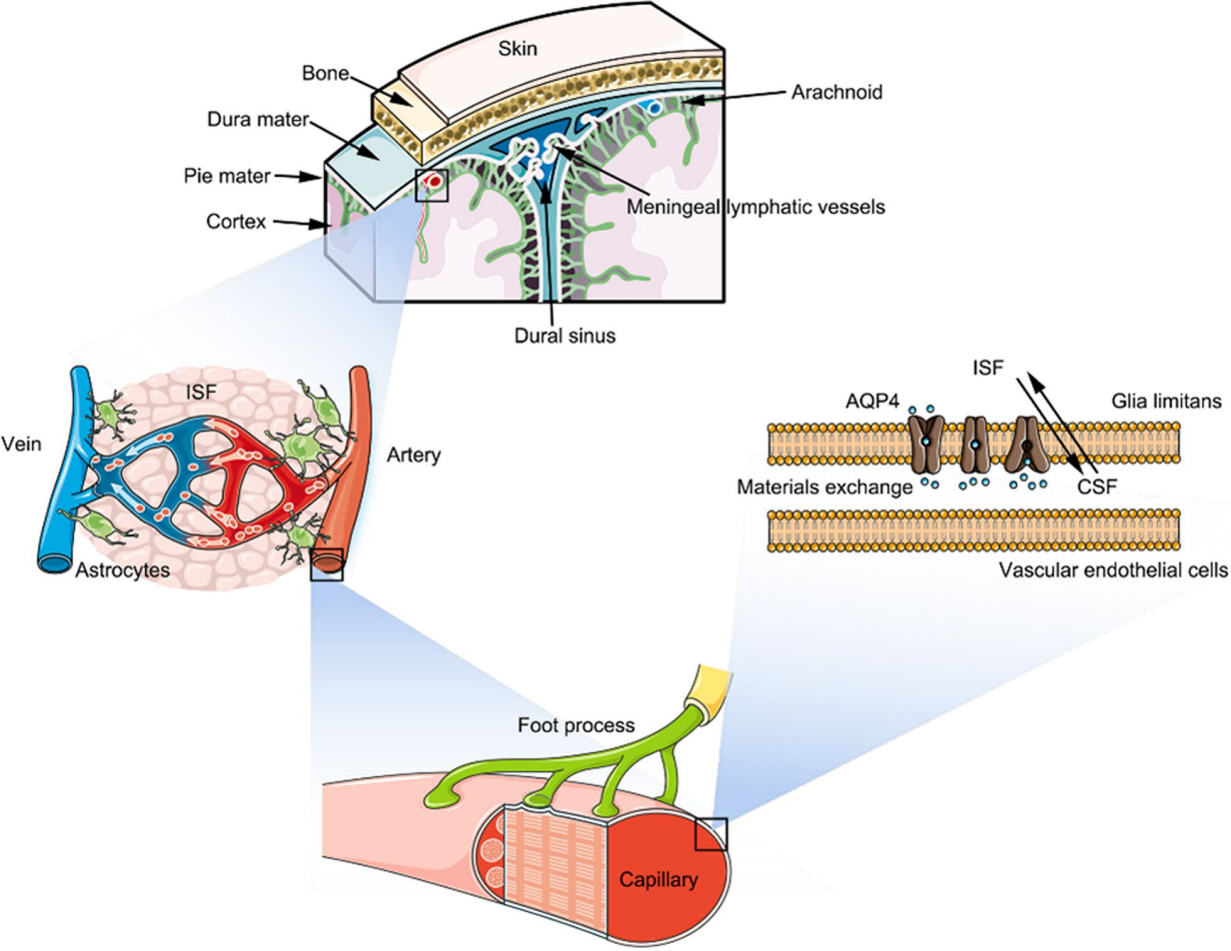
The glial-lymphatic pathway and lymphatic uptake of brain. The meningeal structure of the brain contains dura mater, arachnoid, and pie mater. The meningeal lymphatic vessels are mainly responsible for immunogen uptake. With the arterial pulsation, CSF in the subarachnoid space enters the deep part of the brain parenchyma along the PVS and rapidly enters the brain parenchyma through the rapid transport by AQP4 distributed on the end foot of astrocytes, then returns through the PVS, and finally enters the dural lymphatic vessels

Neurologist used to describe the failure of brain to reject heterotopic tissue transplantation by the term ‘immunologically privileged’ [10]. Much of this perception was derived from a series of experiments in the 1940s. Peter Medawar described the failure of immune status initiated from brain and attributed this failure to the lack of afferent lymphatic drainage in the brain, which has been disproved recently [4, 5, 11–15]. The current understanding of the immune afferent arm in the brain and cerebral spinal fluid (CSF) circulation can roughly be divided into three stages. First, Virchow and Robin in the 1850s demonstrated the presence of fluid-filled tubular anatomy around parenchymal capillaries, perforated arterioles, and veins. This structure was named the Virchow-Robin space (VRS) or para-vascular space (PVS). Although it has been observed that tracers injected into the subarachnoid space can quickly enter the brain parenchyma along the VRS, researchers have also noted that simple diffusion cannot transport macromolecular substances from interstitial fluid (ISF) to CSF [16, 17]. To solve this problem, Iliff et al. [7, 8] proposed and proved a special aquaporin 4 (AQP4) water channels dependent para-vascular pathway: CSF flows into the brain along PVS and then translocates into the interstitium via AQP4 water channels before exiting along venous perivascular spaces [18, 19]. This ‘glial-lymphatic pathway’ provides a mechanism for substance exchange and signal transmission between the brain parenchyma and interstitium (Fig. 1). Although VRS and the glial-lymphatic system shed light on most of the mechanisms of intracerebral fluid circulation, they did not well explain why CSF can be draining into the extracranial LNs [20]. Therefore, researchers speculated that there might be some functional lymphatic vessels that transport CSF out of the brain.

The concept of lymphatic vessels in the meninges was first proposed by Paolo Mascagni in the late eighteenth century. Two centuries later, Csanda [21] reported the existence of lymphatic connections between the central nervous system and the periphery. However, this had not been recognized until the last decade. The current understanding of cerebral lymphatic drainage is mainly based on two reports with direct evidence of meningeal lymphatic vessels (mLVs) in 2015 [4, 5]. Researchers found that the tracers injected into the brain parenchyma of mice could reach the ipsilateral deep cervical lymph nodes (cLNs). However, after ligation of the efferent lymphatics from the deep cLNs, the dural lymphatic vessels of the mice become engorged [22]. Relevant anatomical evidence was subsequently found: an intradural lymphatic network extending toward the skull base along the main branches of the transverse, sigmoid, anterior, and middle arteries [4, 5, 22]. To further verify this result, Mesquita first injected a vascular injury drug into the cisterna magna of mice to disrupt mLVs. Then, he injected tracer into the brain parenchyma and found that small amounts of tracer can reach cLNs [22]. Data obtained from experiments in rodents suggested that lymph fluid containing immune cells and antigens can flow along PVS and/or glial-lymphatic pathways and eventually flow into the cLNs via mLVs [4, 5, 7, 8, 23, 24]. These findings indicate that mLVs are the main route of CSF to the extracranial LNs in the brain.

The mLVs are lined by differentiated lymphatic endothelial cells and are thinner compared to peripheral lymphatics [25]. Based on the anatomical features of the brain, mLVs can be divided into dorsal mLVs along the superior sagittal sinus and transverse sinus course and basal mLVs along the petrous sinus and its sigmoid sinus course. The former has a stronger ability to mediate the transport of inflammatory factors, T cells, and dendritic cells (DCs), in which the direction of lymphatic flow is opposite to that of venous flow in the superior sagittal sinus [23, 26]. The latter contains abundant vasculature and clear lymphatic valves [15, 27]. After injection of fluorescent tracers into the CSF, some sites in the meningeal lymphatics adjacent to the transverse sinuses immediately take up the tracer from the CSF, and these ‘hot spots’ are the first sites for lymphatic uptake of the CSF [15]. Magnetic resonance imaging (MRI) and fluorescence imaging show that when the transverse sinus splits into the sigmoid sinus and petrosquamous sinus, the complexity and the capacity for fluid uptake of these lymphatic structures are significantly enhanced, indicating that basal mLVs are the major roles of CSF drainage [28]. In general, the glial-lymphatic system, mLVs, and cLNs constitute a novel intracranial-to-peripheral drainage pathway (Fig. 1).

### The BBB permitting peripheral T cells infiltration

Challenges for the immune efferent arm of the brain have historically been attributed to the existence of the BBB. Structurally, the BBB consists of a bio-membrane between vascular endothelial cells and glial cells [29–31]. Functionally, the BBB is a dynamic network between peripheral circulation and the brain, preventing the diffusion of large, hydrophilic molecules or organisms and allowing the influx of small, hydrophobic molecules [32]. Therefore, the vast majority of chemotherapy agents cannot cross the BBB to enter the brain. In the past decades, only temozolomide (TMZ) has achieved clinical success. Meanwhile, the BBB only permits limited immunity in the brain since it allows immune cell passage only at the level of the post-capillary venules [32]. However, new pieces of evidence reveal the immunosurveillance of the CNS and the entrance of T cells in the brain, disproving the complete block effect of the BBB on brain immunity [32, 33]. Similarly, highly invasive and diffused brain tumors can destroy the local BBB, leading to drug entry and the infiltration of immune cells [34].

**Fig. 2:**
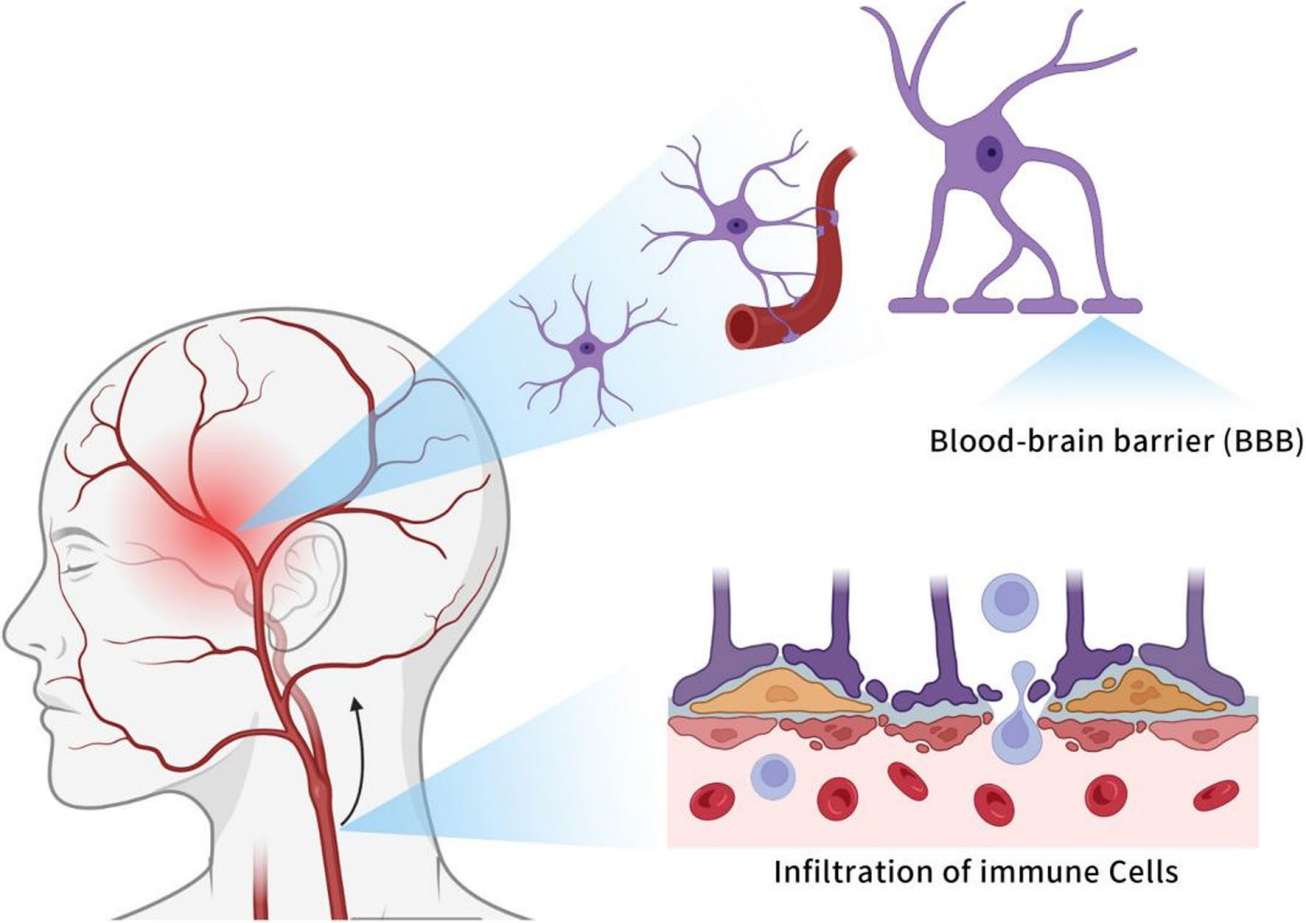
Lymph node released immune cells cross the blood–brain barrier. The The BBB consists of a biomembrane between vascular endothelial cells and glial cells. Under physiological conditions, the BBB allows a small number of immune cells to enter the brain via the post-capillary route

The current understanding of immune afferent and efferent arms breaks the established view of the brain’s ‘immunologically privileged’ zone, theoretically connecting the CSF circulation, mLVs, cLNs, and BBB to form a complete brain immune circuit. LNs are important hubs in the brain immune circuit, communicating intra- and extracranially, and have shown great potential and prospects in brain-targeted drug delivery (Fig. 2).

## The rationale and practice of LNs targeted drug delivery in GBM

### Enhancing afferent signaling promotes T cells infiltration

Despite the complete afferent and efferent arms of the brain, a vast majority of promising immunotherapy strategies, such as immune checkpoint blockades (ICB), have not been translated into clinical success yet. One important reason is that, although ICBs remarkably reduce the ratio of exhausted T cells, the infiltration of effector T cells in the GBM microenvironment remains at an extremely low level [35, 36]. This may owe to the lack of neoantigens that are presented to T cells with high quality [36, 37]. Interestingly, when exogenous vascular endothelial growth factor-C (VEGF-C) was injected into ventricle to promote proliferation of mLVs, endogenous immune responses were remarkably potentiated [27, 38–40]. In preclinical models, T cell numbers increased in mice with VEGF-C-mRNA treatment, which is consistent with the idea that antigen-specific T cells proliferated only after an increase in tumor antigens drained from the CNS [27, 40]. The increase in lymphatic drainage is necessary to present more neoantigens to cLNs and to lead to the enhancement of anti-tumor responses. Therefore, enhancing the presentation of neoantigens from brain to periphery through mLVs and cLNs is important for activating stronger immune responses against GBM.

**Fig. 3:**
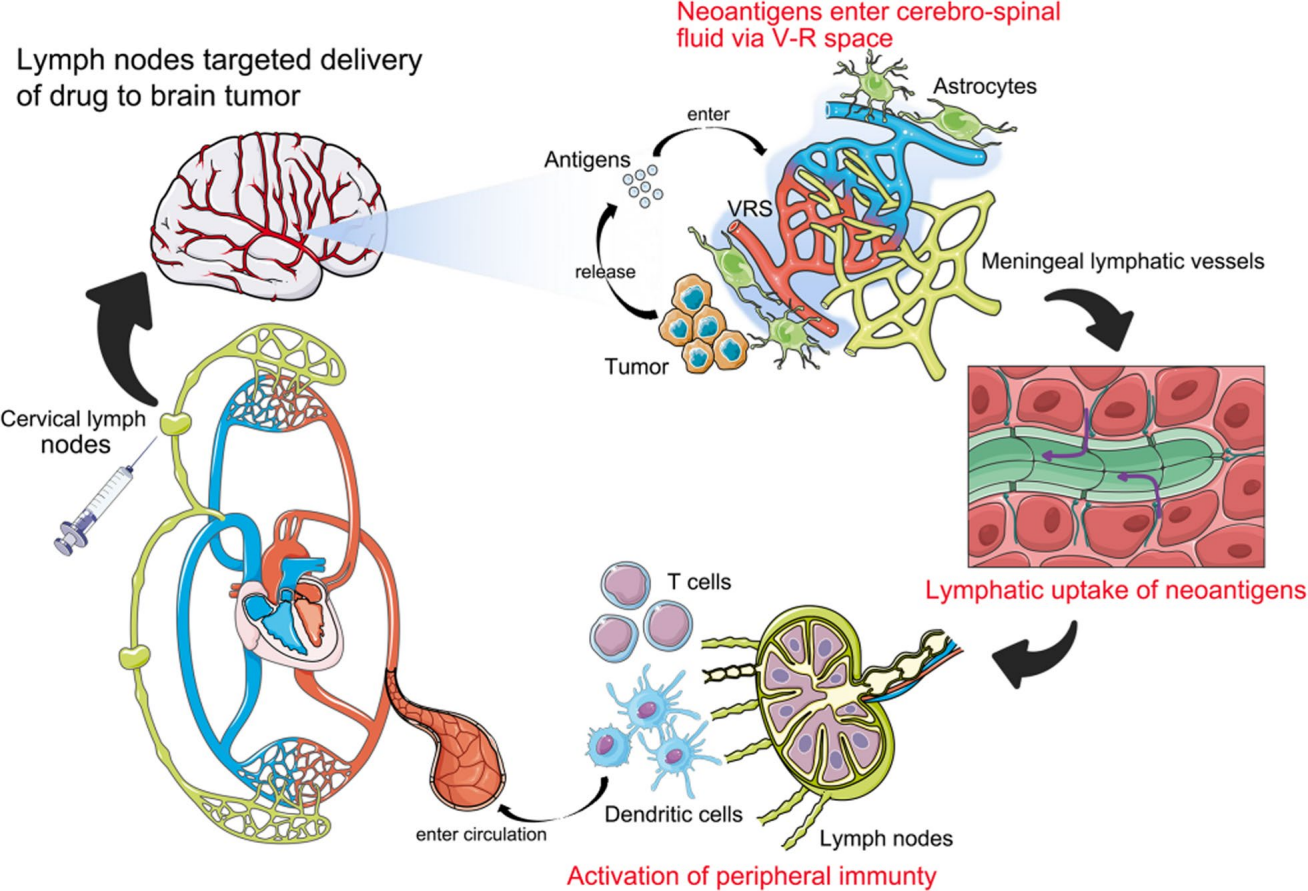
LN targeted drug delivery optimalizes the antigen presentation process. The neoantigens released by tumor were taken by mLVs via the glial-lymphatic pathway. Subsequently, neoantigens were drained to the cLNS and activated the APC and naive T cells within them. As a contrast, LN targeted drug delivery can bypass the above complex links, directly and efficiently activate APC and T cells, and greatly enhance the immune response

From the above findings, the enhanced T cell response is essentially due to the LNs receiving more afferent intracranial tumor-associated immune signals. Thus, if we directly prime LNs with strong neoantigens, can similar immune responses be induced in GBM? Recently, the success of COVID-19 vaccine, considered the hope for ending the worsening coronavirus pandemic, highlighted the value of mRNA vaccine in clinical practice [41]. In cancer, vaccines are also considered as important components of immunotherapy besides ICB and CAR-T. It is worth noting that DCVax-L vaccine (NCT00045968) [9, 42], an autologous tumor lysate-loaded DCs vaccine, has significantly improve patients’ overall survival in both newly diagnosed and recurrent GBM in a recent phase 3 clinical trial. This is a breakthrough in immunotherapeutic strategy in the last decade. The success of DCVax-L indicates that the presentation of individualized patients-derived tumor neoantigens by DCs to T cells might be a crucial step to activate patients’ immunity. Moreover, in consideration of the large amount of DCs and naive T cells in LNs, targeted delivery of tumor neoantigens to LNs to directly activate the immune response appears to be promising with a wide range of applications (Fig. 3).

### Providing alternative pathways to bypass the BBB

According to the above-mentioned study, the increase of tumor lymphatic drainage will stimulate the proliferation of antigen-specific T cells in LNs and eventually lead to an increase in immune cells in tumor microenvironment (TME). In consideration of evidence that T cells can cross the BBB destroyed by tumor cells, the increase in tumor specific peripheral T cells seems to indicate better immune infiltration in TME [34]. However, current passive immunotherapy strategies exemplified by CAR-T, have not yielded phased results in GBM [43–45]. When ex vivo expanded immune cell preparations are reinfused into patients, they should theoretically have a large number of CTLs capable of precisely targeting GBM cells. However, the infiltration ratio of immune cells in TME remains low in clinical practice. We cannot simply attribute this solely to the highly negative immune environment of GBM or an off-target effect. The fact is that the BBB still permits only limited T cell entry, which we consider the unique immunity of the brain [46–48].

The deeper understanding of mLVs reveals a potential bypass that transfers immune cells into the brain. As well known, the basal mLVs contain more abundant vasculature and clear lymphatic valves, mainly responsible for lymphatic drainage and the efferent transmission of immune signals [23]. While the dorsal mLVs that mediate the transport of inflammatory factors and APCs have no lymphatic valves [15, 24, 27]. This finding indicates that immune cells trafficking in the dorsal mLVs may be bidirectional, which explains why the increase of mLVs leads to more CD8^+^ T cells infiltration in TME. More than immunotherapy, conventional chemotherapy also benefits from this potential pathway. Chen et al. [10] developed a polymeric nanoparticle loaded with indocyanine green (ICG). After subcutaneous administration through the neck, the nanoparticles were efficiently pooled in the deep cLNs near the injection site and further transported through the mLVs. The nanoparticles achieved a brain delivery efficiency of up to 8.8%, much higher than the efficiency of intravenous injection reaching the brain. Despite the fact that these studies are mainly performed in preclinical models and the lack of evidence in clinical cohorts, there is no doubt that cLNs’s targeted drug delivery strategy could be a breakthrough in the treatment of GBM with the advantage of bypassing the BBB. In particular, recent research on mLVs mainly began in 2015, so part of their functions remain poorly understood. A deeper and more comprehensive understanding of mLVs and cLNs is urgently required to better evaluate the value of LNs targeted drug delivery strategy in GBM.

## The carrier designed for LNs targeted drug delivery

### Polymeric nanoparticles-based delivery systems

**Fig. 4:**
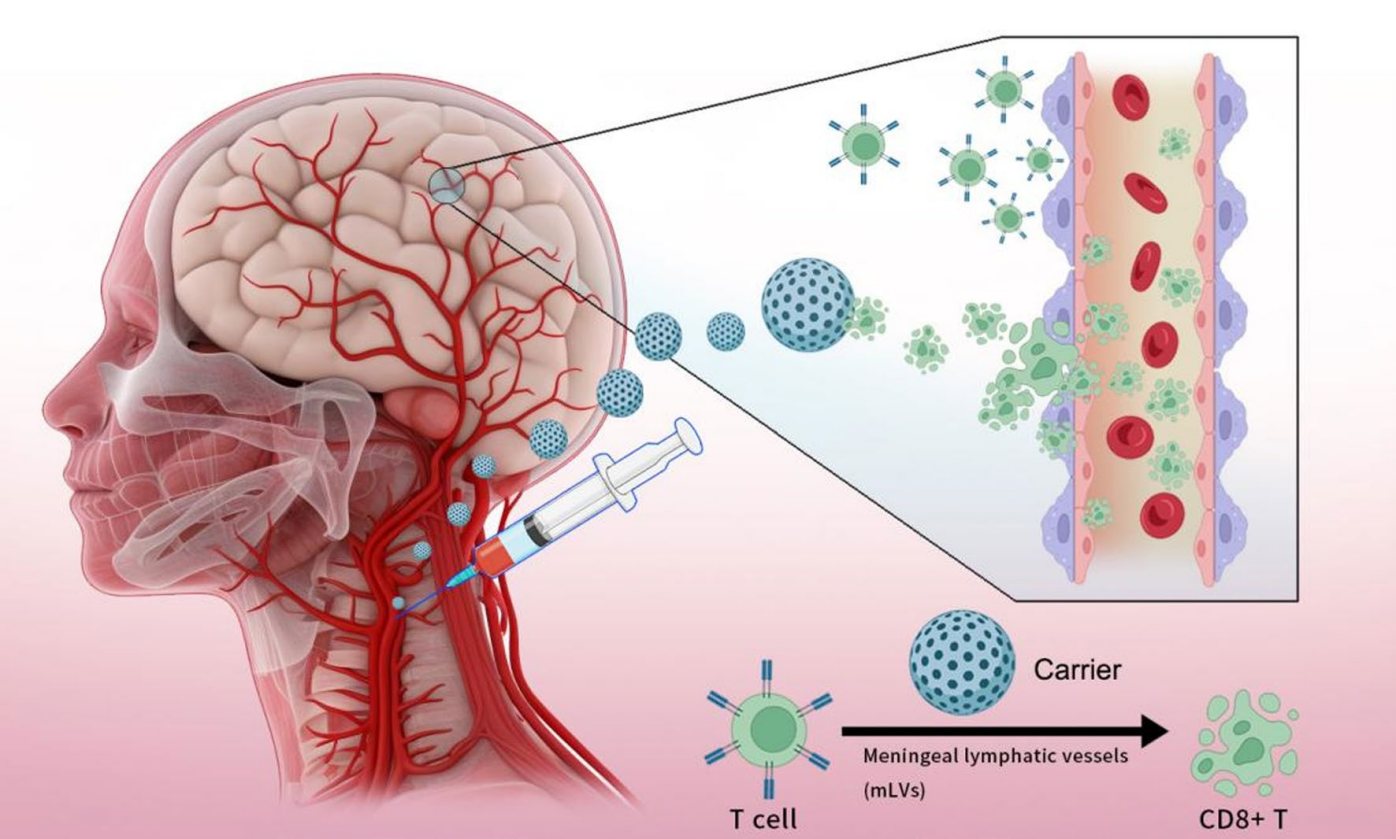
LN-targeted drug delivery activates immune cells and promotes T cell movement into the brain. Through cLNs injection, neoantigen-loaded carriers stimulate the APCs and effector T cells in lymph nodes. Subsequently, the activated T cells enter brain through lymphatic vessels or artery

Polymeric nanoparticles (NPs) delivery systems consist of nano-solid particles, nanogels, micelles, polymer vesicles, and core–shell nanoparticles based on natural or synthetic polymeric materials [49–53]. They are well studied in brain diseases as drug carriers for crossing the BBB. In addition to their strong ability to penetrate biological barriers, their surfaces are also rich in modifiable chemical groups that can be modified to elicit effective immune responses [54]. A number of NPs have showed great biocompatibility and mild side effects in experimental and clinical practices [55]. Thus, polymericas NPs were considered promising carriers for LNs targeted drug delivery (Fig. 4).

The size, molecular weight, charge, shape, and modified ligands are important factors in affecting cancer drug targeting and retention in LNs [56]. Lymphatic vessels absorb the administered agent from the interstitium. Molecules with a hydrodynamic diameter of < 50 nm can be most effectively absorbed, while particles (< 20 nm) are easier to return to peripheral blood [57, 58]. Besides, due to the interaction with hyaluronic acid filled in the interstitium, positively charged NPs are much more difficult to be taken up by lymphatic vessels compared with negatively charged ones [59]. Moreover, NPs modified with ligands targeting LNs can significantly increase lymphatic uptake. For instance, lymphatic vessel endothelial hyaluronan receptors expressed on lymphatic endothelial cells, peripheral lymph node addressins (PNAds) expressed on high endothelial venules (HEVs), and mannose receptors on lymphatic endothelium can facilitate drugs targeting LNs. The addition of a nitrogen-containing six-membered ring to a polymeric structure can promote NPs delivery via proton sponge effect and activate a stronger immune response via the stimulator of interferon genes (STING) pathway [60–63].

To further promote the delivery efficiency of drugs to LNs, Schudel et al. [64] developed a two-stage delivery platform (OND-NP) based on thiolated poly (propylene sulfide) (PPS) nanoparticles and oxaboranediene (OND) linkers. NP are enriched in superficial lymph nodes after subcutaneous injection and spread to deep lymph nodes after the degradation of OND. Karabin et al. [65] also established a hydrogel loaded micellar nanocarrier that was degraded by photooxidation within a month and continuously deliver the drug to the LN resident immune cells. Overall, the polymeric NPs show great capability to target and deliver drugs to LNs and/or to improve adaptive immune response. The application of polymeric NPs to penetrate the inherent barrier has confirmed their potential in a variety of diseases, but their biological safety and cost are still the problems that prevent their clinical development.

### Lipid-based materials delivery systems

Liposomes are an artificially prepared drug delivery system capable of encapsulating the active ingredient within a lipid bilayer to form a mini globular carrier [66, 67]. Their half-life in the circulatory system can be extended by covering the surface with inert polymeric molecules such as oligosaccharides, glycoproteins, polysaccharides, and synthetic polymers [68]. Liposomes are generally used to construct novel vaccine adjuvant delivery systems that protect the long-lasting, slow release of pathogen antigens, a key component in vaccines, and enhance the immunogenicity of vaccines. Studies have shown that some liposomes have unique immunostimulatory functions on their own and are able to induce a broad spectrum of acquired immunity under special conditions [67, 69]. In summary, the lymph node targeted liposomes benefit from the development of microfluidic technology, making the manufacturing process relatively easier, though still face challenges in how to increase the stability and sustained release of liposomes to promote a stronger immune response.

With the development of nanoscience and technology, lipid nanoparticles (LNPs) have been widely studied. LNPs are a new type of lipid drug loading system and one of the most advanced nucleic acid delivery platforms to date. The first RNA interference (RNAi) drugs also took advantage of LNPs as carrier [70–72]. LNPs are multicomponent systems, typically spherical vesicles composed of ionizable lipids or cationic lipidoid compounds, auxiliary lipid cholesterol, protective agent polyethylene glycol (PEG) lipid conjugates, and gene-based drugs or vaccine that enable the entry of gene carrying drugs into cells [73]. Gene drugs or vaccines are encapsulated to avoid degradation, which would greatly enhance the bioavailability of the loaded drugs and reduce the requirement for dosage [74–76]. To date, mRNA vaccine induced hepatic damage has been reported in a series of recent studies [77–79]. The development of LNs-targeted mRNA vaccines holds promise for avoiding side effects on liver. Chen et al. [80] reported one LNs-targeting mRNA vaccine based on LPs named 113-O12B for cancer immunotherapy. The targeted delivery of the mRNA vaccine elicits robust CD8^+^ T cell responses. Moreover, Yu et al. [81] developed self-assembling melittin-lipid nanoparticles (α-melittin-NPs) that are loaded with extra tumor antigens and promotes whole tumor antigen release and activation of APCs in LNs. Although LNPs have demonstrated their potential for clinical applications, they still suffer from a number of drawbacks. The main questions are how to reduce the toxicity of adjuvants and improve the stability and targeting of LNPs.

### Cell-based delivery systems

Currently, applying DCs as vaccine carriers towards LNs has becoming an attractive approach to bypass the delivery and internalization of intrinsic DCs [82, 83]. Their application shows great capability in overcoming several limited factors in antigen presentation and internalization, such as antigen uptake, lysosomal escape and the translation of mRNA antigens. DC vaccines loaded with autologous tumor antigens can induce a potent immune response and are easy to achieve in individualized therapy for patients [84]. Nowadays, HybriCell and CreaVax-RCC have been approved for clinical practice, and a number of other DCs vaccines are in the stage of clinical trials. Among them, the DCVax-L vaccine is the most noticeable one since it significantly improves patients’ overall survival in both newly diagnosed and recurrent GBM in a recent study, which was the only one phase 3 clinical trial with a positive result ever reported in brain tumor immunotherapy [9, 42]. The success of DCVax-L highlights the broad applications foreground for LNs targeted vaccines in tumor therapy. However, the clinical trials of DCVax-L have a large time span and are not purely prospective study. Study also revealed that only 5% of intradermal DCs vaccines actually migrated to lymph nodes [85, 86]. More regrettably, the preparation process of the DCs vaccine involves multiple biotechnology approaches with certain technical bottlenecks, which is more time-consuming and expensive than traditional vaccines. Therefore, despite the approval of the DCs vaccine, there is still a long way to go before it is applied in a wider scope.

### Virus-based delivery systems

Over the past decades, viruses have shown great capability in integrating foreign genes into the host genome and promoting their long-term stable expression. In contrast to the carrier described above, viruses used in LNs targeted delivery are mRNA specialized. Viruses used as carriers are antigenic without toxicity and are able to elicit specific immune responses. Especially nowadays, the COVID-19 vaccine is considered the only hope for ending the worsening coronavirus pandemic [41]. Among a variety of viruses, adenovirus (AV) has been widely used in gene transfer and vaccine loading, with the advantages of easy operation and high infection efficiency [87–89]. Currently, a novel AV loaded coronal vaccine (Ad5-nCov) has been approved for clinical use in China, Mexico, and Pakistan [88]. Undoubtedly, the great advantage of viral carriers is that they can be obtained from in vitro cell culture with low production costs and easy industrialization [90, 91]. Relying on advanced technologies, viral carriers can serve as a universal vaccine platform for efficient delivery of tumor antigens. However, there are still some limitations, exemplified by the off-targeting of viral carriers, which may lead to side effects in normal tissue [91]. Though, from the perspective of economy and convenience, a virus loaded RNA vaccine has greater potential in the treatment of brain tumors. Thus, improving the targeting of LNs by viral carriers facilitates their further applications in drug delivery.

## Conclusion and outlook

Over the past decades, our understanding of brain immunity has evolved from ‘immunologically privileged’ to ‘unique immunity’. Owing to this, a series of experimental immunotherapeutic strategies with great promise have been developed to treat brain diseases. However, most of them have not been translated into clinical success in GBM. These clinical failures are commonly attributed to several intrinsic properties of GBM, including immune desert microenvironment, high heterogeneity, adaptive resistance to therapy, and the BBB blockades [36, 37]. As important hubs of communication between inside and outside the brain, the discovery and deep understanding of the glial-lymphatic pathway and mLVs are significant for researchers. New perspectives reveal the sites of material exchange between ISF and CSF, drainage pathways for cerebral lymph, and channels of communication between the intra- and extracranial lymphatic system. More importantly, they highlight the important roles that cLNs have in brain immunity and pave the way for potential applications of LNs targeted therapy in GBM.

The success of DCVax-L [9] triggered our thinking about the failure of CAR-T: whether there are sufficient amount of T cells in the periphery is not important, and it is important how many of them are able to enter the tumor microenvironment. LNs targeting offers us another possibility: the communication between mLVs and cLNs provides a potential route that can bypass the BBB and promote T cells infiltration into GBM. The study that VEGF-C promotes mLVs proliferation significantly enhances CD8^+^ T cells mediated immune responses in GBM, strongly supports this viewpoint [27, 40]. Current chemotherapy may also be boosted by this pathway, exemplified by the successfully delivery of drug loaded NPs into GBM via subcutaneous administration through the neck [10].

Actually, despite the fact that carriers designed for LNs targeted drug delivery are becoming more mature with the advances of technology, the most crucial difficulty in the treatment of GBM has never been the development of more targeted materials. What is more important is that how to apply LNs targeted carriers into clinical practices. Current understanding of mLVs, CSF circulation pathway, and cLNs is mainly based on rodent models. Most in vivo experiments have been performed in preclinical models as well. Therefore, although LNs targeted drug delivery strategy is of great promise in management of GBM, understanding of mLVs and intracranial lymphatic drainage pathway in human species is still not sufficient. Future work needs to be validated in more human-like models.

## Data Availability

Not applicable.

## Abbreviations

LNs: Lymph nodes
APCs: Antigen present cells
CNS: Central nervous system
GBM: Glioblastoma multiforms
BBB: Blood–brain barrier
DCs: Dendritic cells
CSF: Cerebro-spinal fluid
VRS: Virchow-Robin space
PVS: Para-vascular space
ISF: Interstitial fluid
AQP4: Aquaporin 4
mLVs: Meningeal lymphatic vessels
cLNs: Cervical lymph nodes
MRI: Magnetic resonance imaging
TMZ: Temozolomide
ICB: Immune checkpoints blockade
VEGF-C: Vascular endothelial growth factor-C
TME: Tumor microenvironment
CAR-T: Chimeric antigen receptor T cell
NPs: Nanoparticles
HEVs: High endothelial venules
PNAds: Peripheral lymph node addressins
STING: Stimulator of interferon genes
LNPs: Lipid nanoparticles
AV: Adenovirus
